# Population divergence and gene flow in two East Asian shorebirds on the verge of speciation

**DOI:** 10.1038/s41598-019-44996-5

**Published:** 2019-06-12

**Authors:** Keren R. Sadanandan, Clemens Küpper, Gabriel W. Low, Cheng-Te Yao, Yue Li, Tao Xu, Frank E. Rheindt, Shaoyuan Wu

**Affiliations:** 1 National University of Singapore, Department of Biological Sciences, Singapore, 117558 Singapore; 2 Max Planck Institute for Ornithology, 82319 Seewiesen, Germany; 3 Monash University, School of Biological Sciences, Clayton, VIC 3800 Australia; 4 Endemic Species Research Institute, High-altitude Experimental Station, Chi-chi, 55244 Taiwan; 5 Jiangsu Normal University, School of Life Sciences, Jiangsu Key Laboratory of Phylogenomics and Comparative Genomics, Xuzhou, 221116 China; 6 Tianjin Medical University, School of Basic Medical Sciences, Department of Biochemistry and Molecular Biology, 2011 Collaborative Innovation Center of Tianjin for Medical Epigenetics, Tianjin Key Laboratory of Medical Epigenetics, Tianjin, 300070 China

**Keywords:** Evolution, Genomics

## Abstract

Genetic isolation of populations over evolutionary time leads to the formation of independent species. We examined a pair of shorebirds – the Kentish Plover *Charadrius alexandrinus* and the enigmatic White-faced Plover *C. dealbatus* – which display strong plumage differentiation, yet show minimal genetic divergence based on previous mitochondrial and microsatellite work. Two scenarios may lead to this situation: (1) they represent clinal or poorly diverged populations with limited genomic differentiation despite substantial plumage variation, or (2) they are diverging taxa at the cusp of speciation, with ongoing limited gene flow obliterating signals of differentiation in traditional genetic markers. We compared the genotypes of 98 plovers (59 Kentish Plovers, 35 White-faced Plovers and 4 genomic hybrids) sampled in eastern Asia and Europe using ddRADSeq to harvest over 8000 genome-wide SNPs. In contrast to previous studies, our analyses revealed two well defined genomic clusters, with limited hybridization and a narrow contact zone. We also uncovered significant differences in bill length and further sex-specific differences in size, which may signal differences in mate choice between Kentish and White-faced Plovers. Our results support the hypothesis that this shorebird duo is on the verge of speciation.

## Introduction

“*Firstly, why, if species have descended from other species by insensibly fine gradations, do we not everywhere see innumerable transitional forms? Why is not all nature in confusion, instead of the species being, as we see them, well defined?*” – Darwin (1859)^1^

Speciation is the evolutionary process by which populations differentiate to become distinct species. The formation of barriers to gene flow plays an important role in the speciation process^2^, with allopatric barriers being its most well recognized driver^3^. However, there is now growing recognition that variation in intensity of sexual selection or preferences for different traits may also lead to species divergence^4,5^. Speciation can occur extremely rapidly over the course of a few generations^2,6^ or over long time scales, and may or may not be accompanied by gene flow from overlapping congenerics^7^. Consequently, young species can often be difficult to distinguish from divergent populations^8–10^. Biologists often conservatively use the term ‘taxon’ to refer to difficult cases in which there is no consensus on whether a lineage qualifies as a full species or is merely a divergent population.

Shorebirds (order Charadriiformes) are an old avian lineage with high behavioural and morphological diversity^11–14^. *Charadrius* plovers, one of the core clades and the name-sake group, have been an important study subject in evolutionary and ecological research given their unusual diversity in mating and parental care systems, even within a single species^15,16^. This variation in breeding behaviour has also been linked to population differentiation and diversification, whereby monogamous breeding systems in plovers are thought to be key promoters for population divergence whereas polygamous behaviour may instead slow down divergence^5^. The advent of Next-Generation Sequencing (NGS) technologies provides us with powerful tools to investigate differentiation across plovers from a genomic perspective^17^.

**Figure 1:**
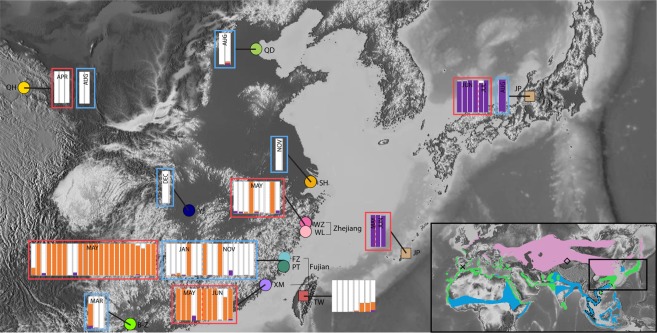
Map of sampling locations and _STRUCTURE_ results at *K* = 3 for eastern Kentish and White-faced Plover populations. The inset shows global distribution of Kentish and White-faced Plovers^22^: green areas demarcate year-round resident populations of Kentish Plovers, purple shows breeding ranges of migratory populations and blue areas show wintering regions; the thin dotted line (• • •) outlines the presumed breeding range of White-faced Plovers and the stippled line (−) demarcates their known wintering range. Cross and open diamond refer to sampling localities in Spain and far western China respectively. Samples collected during the breeding season (May–July) are outlined in red whilst those collected during the non-breeding season are outlined in blue. Sampling dates for Taiwan are unknown and thus lack outlines. Sampling locality acronyms are as follows: QH – Qinghai, QD – Qinhuangdao, JP – Japan, SH – Shanghai, YJ – Yuanjiang, WZ – Wenzhou, WL – Wenling, FZ – Fuzhou, PT – Putian, XM – Xiamen, TW – Taiwan, B-Z – Beihai-Zhanjiang. Map modified from https://maps-for-free.com/ (© OpenStreetMap contributors) using Adobe Illustrator CC v.23.0.1 (https://www.adobe.com/products/illustrator.html).

Despite the status of *Charadrius* as an extremely well-studied bird genus, the last few years have seen the discovery of novel populations and taxa^18^. Among these, Kennerley *et al*.^19^ documented the discovery of a distinctly-plumaged East Asian population of the Kentish Plover *Charadrius alexandrinus* complex that was shown to refer to the generally synonymized taxon name *dealbatus*. Several aspects regarding the breeding plumage of this rediscovered taxon, such as the extent of white on the face, and minor differences in morphology, render it as distinct as many universally recognized plover species^19,20^, which has led to its occasional recognition as an independent species named White-faced Plover *C. dealbatus* (e.g.,^21^). However, a first molecular inquiry using mitochondrial DNA and microsatellites on museum specimens found no evidence of genetic differentiation between White-faced and Kentish Plovers^20^. While the two taxa overlap widely in their Southeast Asian winter distribution (Fig. 1), the breeding grounds of the enigmatic White-faced Plover have never been characterised in detail, although they have been found to breed along the southern coastline of China and northernmost Vietnam^22^. It is therefore unclear to what extent the breeding range of White-faced Plovers overlaps with the breeding range of Kentish Plovers.

**Figure 2:**
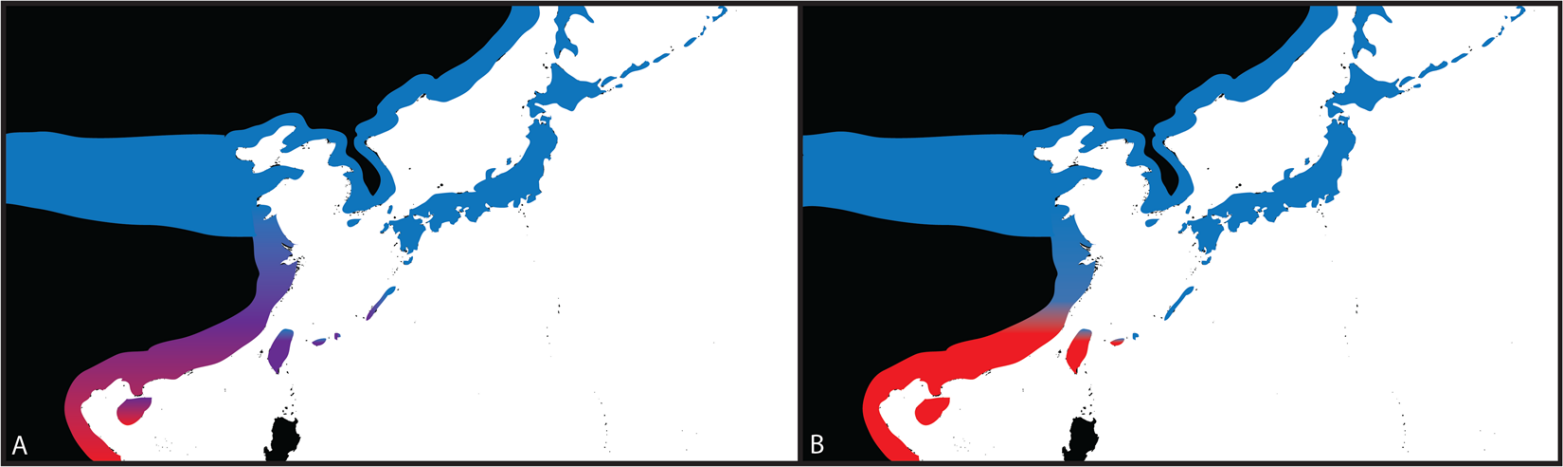
Two hypotheses for the interaction between Kentish and White-faced Plovers: (A) shallow divergence with widespread gene flow - a genomic cline from northern to southern China, with the greatest differentiation seen at either end of the spectrum of distribution and a broad hybrid zone, and (B) deeper divergence with limited gene flow – the two taxa meet at a narrow contact zone and show limited hybridization.

The differentiation in breeding plumage between White-faced and Kentish Plovers in the absence of mitochondrial and microsatellite differentiation has posed a conundrum that is difficult to reconcile. On the one hand, these may represent two populations with poor genomic divergence but substantial plumage differentiation. Under this scenario, we would expect a broad genetic cline across geographic populations (Fig. 2A). On the other hand, they may represent two deeply differentiated subspecies or even young incipient species whose divergence in certain traits does not track their level of genetic diversification, possibly because of continuing low levels of gene flow and introgression. In this case, instead of observing genomic clinality and gradual changes between the two breeding populations, we would find a narrow contact zone of overlap and gene flow (Fig. 2B). Under either scenario, the most important arena in which to study gene flow would be the central to southern coastline of China, an area suspected to be the overlapping breeding zone for both Kentish Plovers and White-faced Plovers.

Here we investigate the two possible scenarios by examining the patterns of genomic differentiation between plovers along the east coast of China. We used a genotyping-by-sequencing approach^23^ to harvest over 8000 genome-wide SNPs across 98 individuals (~60% Kentish Plovers, ~40% White-faced Plovers). Our taxon sampling covers a broad geographic range to investigate the magnitude of differentiation and dynamics of the contact zone, and includes a densely sampled coastal transect in China, a population from the western distribution end of Kentish Plovers in Spain, and a population from another East Asian subspecies *nihonensis* that inhabits island archipelagos at the northeastern end of the Kentish Plover breeding distribution. Our sampling incorporates a comprehensive dataset of body measurements to explore whether biometric changes correlate with the genomic clines. Our objective is to establish the nature of genomic connectivity between these two taxa to ascertain how and if two such divergent plumage phenotypes can be maintained in the presence of gene flow.

## Results

### Population genetic structure and hybridization

We recovered a total of 1,729,307,240 paired-end 150 bp reads from Illumina sequencing of 122 plover samples. Preliminary filtering of the sequencing data included manually removing seven individuals with poor sequencing coverage (less than one million reads). Each of the remaining 115 samples had a mean sequencing depth of 139x. A further nine individuals were removed in PLINK 2.0 as they exhibited more than 30% missing stack data, whilst eight additional individuals that showed evidence for first or second-order kinship with another individual were also pruned. This yielded a final dataset of 98 individuals and 8088 SNPs. The total genotyping rate was 0.990466. Of these 98 individuals, 59 were Kentish Plovers, 35 were White-faced Plovers and four consistently displayed genomic signatures intermediate between the two taxa in both _STRUCTURE_ and PCA (see below).

**Figure 3:**
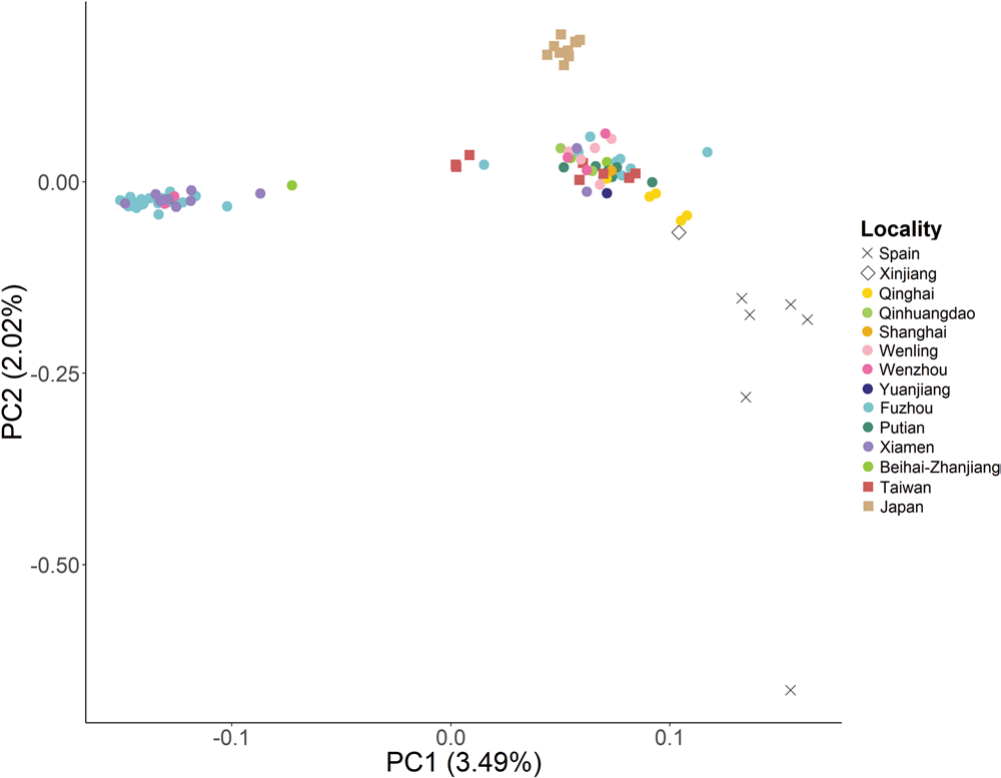
Principal component analysis of the complete final dataset of 98 individuals using 8088 SNPs, explaining 5.51% of the total variance observed. Colours and shapes of locality symbols match those shown in Fig. 1.

Our population genetic analysis using _STRUCTURE_ showed a primary division between Kentish Plovers and White-faced Plovers, and subsequent division between allopatric subspecies *alexandrinus* (the Chinese mainland and Taiwanese samples) and *nihonensis* (Japanese samples, Figs 1, 3 and S2). Our Netview results mirrored this pattern, with White-faced Plovers forming a tight cluster and Kentish Plovers forming a looser cluster, and with Japanese and Spanish individuals outside the main Kentish Plover cloud (Fig. S1). Our result for the Japanese samples is consistent with the traditional recognition of the Japanese Kentish Plover population as an independent subspecies *Charadrius alexandrinus nihonensis*, characterized by a longer bill and slightly duller breeding plumage^24^.

For the remaining samples, individual cluster assignments showed a sharp population genetic break from north to south along the eastern Chinese coastline, with a narrow zone of overlap around the border between the provinces of Fujian and Zhejiang. The majority of birds sampled in Fujian were White-faced Plovers (32 out of 52 individuals, or 62%) and the minority were Kentish Plovers (19 out of 52 individuals, or 37%). More importantly, when we considered only records from the breeding season (April–July, the percentage of White-faced Plovers in northern Fujian increased to 83% (29 out of 35 individuals). Conversely, in southern Zhejiang, 78% of breeders were Kentish and only 22% were White-faced Plovers (7 and 2 individuals respectively; Fig. 1). A single individual in northern Fujian emerged as a genomic hybrid between the two clusters (Fig. 3).

In Taiwan, a large island east of Fujian, five of the eight (62.5%) plovers sampled were Kentish Plovers (Fig. 1). The remaining three individuals had genotypes consistent with hybrids between Kentish and White-faced Plovers (Fig. 3).

The results of the Principal Component Analysis (PCA) were consistent with the _STRUCTURE_ results. White-faced and Kentish Plovers separated along PC1, with White-faced Plovers forming a tight cloud, whereas Kentish Plovers were more broadly dispersed along both axes (Fig. 3). The distribution of Kentish Plovers along PC2 from top to bottom roughly matched a continental East-West cline, with samples from Japan on one end and samples from Spain on the other. Samples from western China (Qinghai and Xinjiang) emerged as intermediate between the coastal eastern Chinese and Spanish samples, further reinforcing the East-West clinal distribution.

Our summary statistics also echoed the _STRUCTURE_ results, with White-faced Plovers displaying high genetic divergence and population differentiation from all Kentish Plover populations. Within the Kentish Plover populations, the Japanese population (*nihonensis*) consistently appeared to be the most distinct, whilst divergences of Spanish and Western Chinese populations mirrored their geographic distance from other populations.

### Morphological differentiation

**Figure 4:**
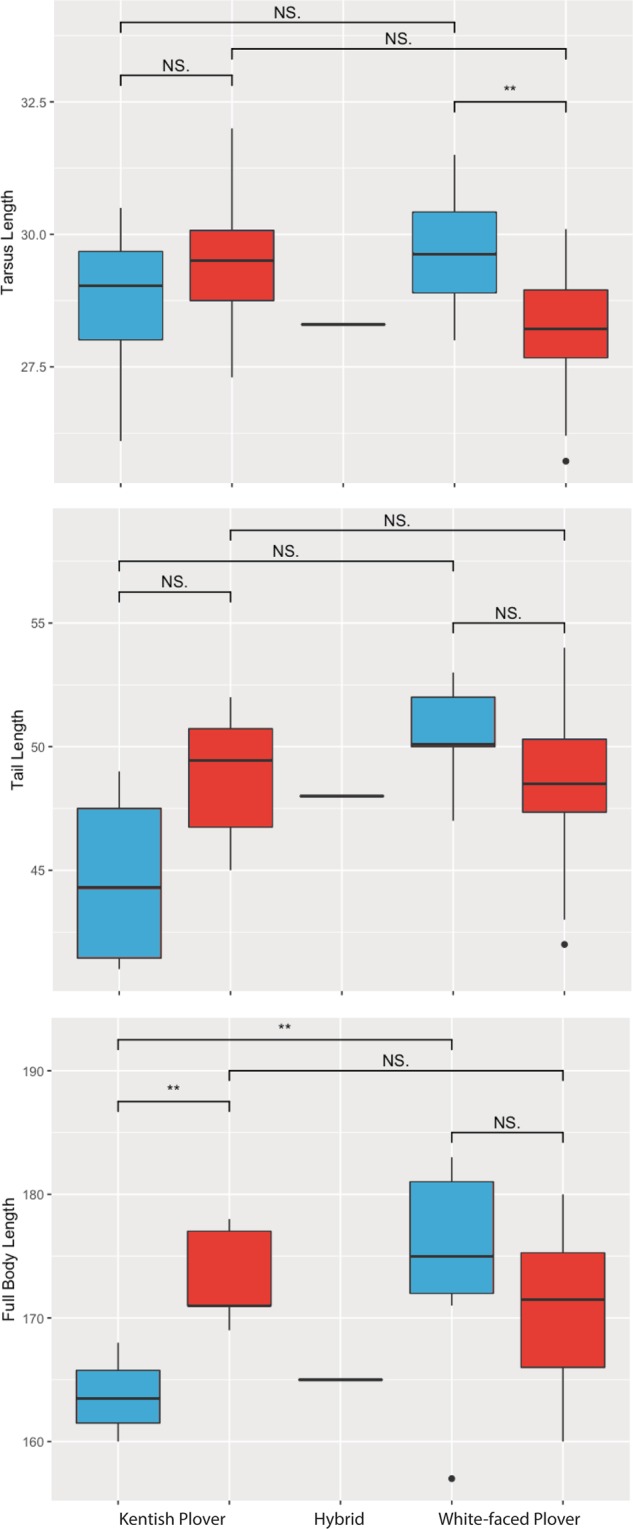
Box plots of morphological traits with significant sex-specific interactions for Kentish (left) and White-faced Plovers (right). From top to bottom: Tarsus length, tail length and full length measurements. Males are depicted in blue and females in red. Significance was calculated using student’s t-test.

The results of all Shapiro-Wilk tests for normality of the morphological traits were non-significant (P > 0.05 for all tests). Two way ANOVAs showed that White-faced Plovers had longer bills than Kentish Plovers (White-faced Plovers: 17.97 ± 0.64 mm [mean ± SD], Kentish Plovers: 16.94 ± 1.10 mm) (${F}_{(\mathrm{1,1})}$= 13.28, P = 0.0008, Table S3), with 27% of the variance in bill length explained by ‘taxon’. Additionally, we found significant species-sex interactions for three other size measurements (Tarsus (${F}_{(\mathrm{1,1})}$= 10.17, P = 0.0029); tail (${F}_{(\mathrm{1,1})}\,$= 4.714, P = 0.0366) and full body length (${F}_{(\mathrm{1,1})}$= 5.95, P = 0.0199): White-faced Plover males were significantly longer than Kentish Plover males in full body length (and had non-significantly longer tarsi and tails), but there was no significant difference between White-faced and Kentish Plover females for all three measurements (Table S3; Fig. 4). The sole hybrid individual for which biometric data were available was female, and exhibited measurements more closely resembling White-faced Plovers than Kentish Plovers (Fig. 4).

## Discussion

The evolutionary history of the White-faced Plover has long been debated by ornithologists and taxonomists alike. On the one hand, this taxon is uniquely distinct in its breeding plumage, with coloration differences that surpass those of many other *Charadrius* plover species^19,20^. On the other hand, evidence from mitochondrial DNA and a set of microsatellites have revealed limited differentiation between White-faced and Kentish Plovers^20,25^. While they overlap widely in their wintering range across Southeast Asia and southern China, their breeding range dynamics are little understood, and it is not known whether their breeding distributions meet in a clinal fashion or if there is a discrete contact zone between the two taxa (Fig. 2).

### Two discrete taxa with limited genomic intermediacy

Genomic analysis of more than 8000 SNPs showed that White-faced and Kentish Plovers are clearly differentiated. This differentiation is at a level higher than that observed between Japanese *nihonensis* and Chinese *alexandrinus* Kentish Plovers in this region, suggesting that *dealbatus* and *alexandrinus* populations have already diverged further (Fig. 1; Fig. 3; Fig. S1). The deepest genomic divisions within our sampling regime were exclusively between White-faced Plovers and Kentish Plovers (*K* = 2 in Fig. 1; PC1 in Fig. 3), even though our geographic sampling encompassed Spanish individuals from the far western end of the Kentish Plover’s breeding distribution roughly 11,000 km from the sampling sites on the Chinese coast. This confirms earlier results^26^ showing that across continental Eurasia, Kentish Plovers exhibit very low differentiation. Our results show that genomic divisions between White-faced and Kentish Plovers from the same areas in Fujian Province (Fig. 1) appear much deeper than those between members of opposing ends of the Kentish Plover continental distribution (Table 1).

**Table 1:** Summary statistics for Kentish and White-faced Plover populations, with ${{\rm{d}}}_{{\rm{xy}}}$ (genetic distance) displayed above the diagonal and ${{\rm{F}}}_{{\rm{ST}}}$ (population differentiation) below the diagonal. Kentish Plover – western China comprises populations from Xinjiang and Qinghai, and Kentish Plover – eastern China comprises all other mainland Chinese populations and Taiwan. Hybrids were excluded from this analysis.

	Kentish Plover - Spain	Kentish Plover - western China	Kentish Plover – eastern China	Kentish Plover - Japan	White-faced Plover
Kentish Plover - Spain		0.026031	0.017603	0.032294	0.034839
Kentish Plover - western China	0.0151		0.014063	029786	0.029611
Kentish Plover – eastern China	0.0186	0.0051		0.01762	0.01768
Kentish Plover - Japan	0.0592	0.0505	0.0382		0.029375
White-faced Plover	0.0868	0.0684	0.0564	0.086	

What is the nature of gene flow dynamics between Kentish and White-faced Plovers in areas of potential sympatry? To answer this question, we relied on fine-scale sampling especially on the coast of northern Fujian and southern Zhejiang provinces, where their breeding ranges come close to each other (Fig. 1). Despite our intensive sampling of 68 genotyped individuals across China’s continental coastline, we only clearly identified a single individual ( < 1.5%) near Fuzhou that exhibited a signature of genomic admixture, with roughly ¼ White-faced and ¾ Kentish Plover ancestry in its overall make-up. All other individuals sampled, including all other 60 birds from Fujian and Zhejiang, had genotypes that were fully assigned to pure Kentish or White-faced clusters based on over 8000 SNPs (e.g., Fig. 1; Fig. 2). This near-dichotomous pattern suggests that although White-faced and Kentish Plovers still may have the capacity to interbreed, they probably only do so infrequently. Such reproductive isolation is typical for young avian sister species meeting along a narrow hybrid zone^27–29^. From our transect data, the area where the breeding ranges of Kentish and White-faced Plovers meet appears to be in southern and central Zhejiang. Between Taizhou and Wenzhou along Zhejiang’s shoreline, our sampling during the breeding season revealed only two White-faced Plovers and seven Kentish Plovers (Fig. 1). Further south, in Fujian, 83% of our 35 samples from May and June were White-faced Plovers (excluding the one hybrid). The majority (16; i.e., 80%) of the 20 samples from non-breeding months (January, March, November) in Fujian and further south were pure Kentish Plovers based on their genomic SNP profiles, while the remainder were White-faced Plovers (Fig. 1). This pattern is consistent with the Kentish Plover’s higher global abundance, and with the notion that Kentish Plovers use the breeding areas of White-faced Plovers during winter and during migration.

On the island of Taiwan, we detected another three out of eight individuals with a similarly admixed profile as the one hybrid from Fujian, with the remainder being pure Kentish Plovers (Fig. 1). This suggests a fundamentally different interaction between these two taxa on Taiwan compared to the mainland, and in agreement with our own observations that White-faced Plovers seem to be a marginally occurring bird on Taiwan’s west coast that is greatly outnumbered by Kentish Plovers (per. obs.). In situations where two hybridizing species co-occur, hybridization is typically promoted by the rarer species^10,30–32^. On Taiwan, consequently, the two plovers may find themselves in a situation that favours hybridization more than on the mainland, where the two seem to hybridize much more rarely. Future investigations into the dynamics of breeding populations on Taiwan will shed more light on this finding.

### Phenotypic differentiation in the Kentish Plover complex

White-faced Plovers are known to differ greatly in breeding plumage coloration and by a number of biometric measurements from other members of the Kentish Plover complex^19,20,25^. In this study, we corroborated these phenotypic differences based on unequivocal genomic assignments and examined further differences between the sexes. Regardless of sex, White-faced Plovers had a significantly longer bill than Kentish Plovers based on bill only and head plus bill measurements (Table S3). This is consistent with other recent results^25^. As for tarsus, tail and full length measurements, we detected a significant sex-specific interaction whereby male White-faced Plovers were larger than male Kentish Plovers, even though female White-faced Plovers were of equal size or even marginally smaller than Kentish Plover females (Table S3; Fig. 4). This phenomenon may hint towards differences in the intensity of sexual selection^16^ or male competitiveness between species^33^, which could play a role in reproductive isolation^34^ or even the dynamics of speciation. Based on observed differences in size, it is plausible that White-faced Plovers displace Kentish Plovers during the breeding season on the southern Chinese coast. Particularly, male Kentish Plovers, with their smaller overall body size (Fig. 4), may be less competitive than White-faced Plovers when it comes to establishing and defending territories.

The White-faced Plover’s distinctly longer bill is also consistent with a continent-wide trend within the Kentish Plover complex towards increasing bill length towards the east. The Japanese subspecies *nihonensis* has been hitherto defined almost solely on account of its distinctly longer beak in comparison with *alexandrinus*, even though there are also differences in breeding plumage^24^. Consistent with previous studies^26^, Japanese Kentish Plovers formed a fairly distinct genetic cluster in both PCA and _STRUCTURE_ analyses and showed high levels of differentiation from samples collected from the adjacent northern Chinese mainland (Fig. 1; Table 1), substantiating their division as an independent subspecies, although their differentiation is much shallower than that of White-faced Plovers.

In the future, comparative behavioural studies should focus on the interactions of the two taxa in the contact zone during the breeding season in order to relate patterns of sexual dimorphism and taxon differences to sexual reproductive traits. In *Charadrius* plovers, breeding systems are known to influence the propensity of a species to differentiate, with polygamous species showing lower population differentiation than monogamous species^5,26^, but great variation in behaviour and plumage characteristics exists even within a single species such as the Kentish Plover^35^.

## Conclusion

We have shown that White-faced and Kentish Plovers are two genomically diverged taxa. Sampling across a transect spanning their continental contact zone and adjacent areas, we identified only few (<1.5%) hybrids among our samples of breeding birds, indicating that gene flow, while present, is rare between these two taxa. Our results favour a narrow contact zone resulting in two lineages with deep genomic differentiation, rather than a genetic cline from one form to the other (Fig. 2B).

Their pronounced differences in breeding plumage coloration are matched by different bill sizes and sex-specific differences in tarsus, wing and full length measurements, hinting at differences in the intensity of sexual selection between the two taxa. Future research into differences in the reproductive behaviour of these two young taxa may shed light on the evolution of sexual traits in these two shorebirds.

## Methods

### Field sampling

We sampled blood from 122 individuals belonging to the Kentish Plover *Charadrius alexandrinus* complex (including White-faced Plover *C. dealbatus*), mainly in China across a coastal transect spanning the breeding distribution of the two forms (n = 87), but also including samples from Taiwan (n = 18), Japan (n = 10) and Spain (n = 7) (Table S1). We captured plovers either using walk-in funnel traps placed on nests or with mist nets. Approximately 30–50 µL of blood were collected from each bird via brachial venipuncture before releasing the bird. We stored blood in ethanol or Queen’s Lysis Buffer^36^ until further processing in the laboratory. Sampling protocols were approved by Jiangsu Normal University and the National Natural Science Foundation of China.

### ddRADSeq library preparation

The following procedures were approved by National University of Singapore’s Office of Safety, Health, and Environment. We extracted DNA either with DNEasy Blood & Tissue Kits (Qiagen) using the manufacturer’s recommended protocol or using an ammonium salt method^37^. We used a Qubit® 2.0 Fluorometer to quantify DNA concentrations of extracts.

We performed double digest restriction enzyme associated DNA sequencing (ddRADseq^38^) as per Tang *et al*.^23^. We split samples into total DNA yield bands between 170–500 ng, based on post-extraction concentrations. We then calculated input volumes for the first restriction step based on these cut-off values. We used the restriction enzymes EcoRI and Msp1 (New England Biolabs Inc.) to double digest the samples for 3.5 hours at 37 °C, followed by clean up using a 1.1X ratio of Sera-Mag SpeedBead Carboxylate-Modified Magnetic Particles (Thermo Scientific). We then quantified samples again and ligated unique barcodes (PIE adaptors) to the fragments using T4 DNA Ligase (New England Biolabs Inc.) at 16 °C for 16 hours.

For subsequent library preparation, we split the samples into seven pools for distribution across two Illumina HiSeq 4000 lanes according to their post-restriction concentrations. We pooled samples with similar post-restriction concentrations to avoid either over or under-representation of individual samples. We cleaned up each pool with AMPure XP beads (Agencourt) using a 1.5X bead ratio, and then carried out size selection for the seven pools using a Pippin Prep Gel Electrophoresis system (Sage Science) to isolate fragments for a sample peak of 420 base-pairs in length. After another subsequent AMPure XP clean up, we amplified size-selected fragments using a polymerase chain reaction (PCR) for 12 cycles, followed by a final AMPure XP clean up step. We screened pools for quality control on a Fragment Analyzer (Advanced Analytical) and then quantified pools on a Qubit 2.0 Fluorometer before creating two final libraries of pools in equimolar proportions. We submitted the DNA libraries to Novogene (Tianjin, China) for 150 bp paired-end sequencing using the Illumina HiSeq X Ten platform.

### Data processing and SNP calling

We used FastQC (Babraham Bioinformatics) to analyze sequence quality across all base positions. Demultiplexing was then performed using the *process_radtags* command in STACKS v1.34^39^. We removed samples with less than one million reads from subsequent analysis. We aligned the sequence reads of the remaining 115 samples against the congeneric Killdeer (*Charadrius vociferus*) genome assembly^40^ using BWA-MEM^41^.

We used the pipeline *ref_map.pl* in STACKS to call single nucleotide polymorphic markers (SNPs), employing a minimum stack depth of 10 in our analyses. We ran the ‘populations’ module to retain loci present in more than 90% of individuals, whilst assigning all individuals to a single population. To reduce the effects of linkage disequilibrium in subsequent analyses, we only accepted one SNP from each locus using the *–write_single_snp* option provided.

We ran PLINK 2.0^42^ to remove any individuals exhibiting more than 30% missing data, and then to further filter SNPs to account for missing data and linkage disequilibrium. We checked for SNPs under selection using BayeScan^43^.

We used the R package, SNPRelate^44^ to estimate pairwise kinship coefficients using maximum likelihood estimation. We removed a single individual from each pair which displayed a kinship coefficient above 0.125 (second order kinship). We then re-ran the *populations* module in Stacks v1.34 and re-ran PLINK 2.0 without filtered individuals/loci to generate the final dataset.

### Population genetic analysis

We assessed population subdivision using a model-based clustering approach implemented in _STRUCTURE_ v2.3.4^45^. First, we removed any singleton or doubleton alleles^46^ using a minimum allele count filter in PLINK 2.0. We then implemented _STRUCTURE_ runs using the remaining 5639 SNPs without *a priori* hypotheses of cluster membership. We ran _STRUCTURE_ from *K* = 1 to *K* = 10 with five iterations per *K*. For each iteration we implemented a burn-in of 100,000 generations and MCMC for 500,000 generations. We used _STRUCTURE_ Harvester Web v0.6.94^47^ to process the output and subsequently averaged the results across replicates by evaluating individual ancestry coefficients (q values) with CLUMPP v1.1.2^48^. Averaged population subdivision over changing values of *K* is depicted in Fig. S2, while the results of *K* = 3, the optimum *K* value as determined by the Evanno method, which calculates the most statistically likely number of genetic clusters^47^, are additionally incorporated into Fig. 1.

We then used Netview, a network theory-based approach, to construct networks of individuals illustrating the connectivity and information flow within and between populations based on genetic similarity^49,50^. Different network topologies were explored by specifying different values of *k*, which dictates the number of genetically nearest neighbors each sample is connected to. We ran Netview for *k* values of 1–35, and plotted the resultant graphs using the Kamada-Kawai force-directed graph drawing algorithm^51^ as implemented in iGraph^52^. We explored population structure using a PCA in SNPRelate^44^ using a genetic covariance matrix calculated from genotypes.

Among our samples we identified hybrids between Kentish and White faced Plover based on their consistent genotypic assignment indicating mixed genotypes based on both _STRUCTURE_ and PCA analyses.

We calculated pairwise population summary statistics for Kentish and White-faced Plover populations using the R packages Hierfstat^53^ and Adegenet^54,55^. We divided our Kentish Plover samples into four populations (Spain, western China, eastern China and Japan) and retained White-faced Plovers as a single population. We excluded any hybrids we identified from this analysis. We utilized Nei’s genetic distance in Adegenet to calculate ${{\rm{d}}}_{{\rm{xy}}}$ and calculated pairwise ${{\rm{F}}}_{{\rm{ST}}}$ using Hierfstat’s ‘Nei87’^56^.

### Morphological analysis

We recorded morphological measurements for a total of 54 birds sampled along the Chinese coastline (Table S2), including the lengths of wings, tarsi, bills, as well as body weight. We measured tarsus lengths from the outer bend of the tibiotarsal articulation to the base of the toes, and recorded bill length as the distance of the tip of the rostrum to the base of the forehead. We did not have access to morphological measurements for many of the samples from other localities, and in cases where we did we chose not to include them to reduce recorder bias from influencing our data analyses.

We obtained ddRADSeq genotypes for 41 of the 54 measured individuals. These comprised 13 males and 28 females, of which we identified 11 birds based on their genotypes as Kentish Plovers, 29 as White-faced Plovers and one as a hybrid (see Results). We assessed normality of the morphological data using Shapiro-Wilk tests^57^. For traits that adhered to a normal distribution we examined differences between sexes and taxa using two-way ANOVAs. We utilized the R package ggplot2^58^ to generate boxplots comparing morphological measurements between the two genomically-prescribed groups for the measurements which showed significant interactions between taxa and sex, and plotted relevant significance bars with the R package ggsignif^59^. We used R version 3.4.1 for all statistical analyses^60^.

### Ethics statement

Research protocols were approved by Jiangsu Normal University and National Natural Science Foundation of China. All lab work was conducted in accordance with regulations outlined by the National University of Singapore’s Office of Safety, Health, and Environment.

## Supplementary information

Supplementary Information

## Data Availability

The Kentish and White-faced Plover genomic data are pending accession on Genbank NCBI. Raw Stacks output and custom scripts are available from the corresponding authors upon request. All other data analysed during this study are included in the Supplementary information files.
