# Supplementary material for: Population divergence and gene flow in two East Asian shorebirds on the verge of speciation
Source: Sci Rep. 2019 Jun 12;9:8546. doi: 10.1038/s41598-019-44996-5 (PMC6561938; doi:10.1038/s41598-019-44996-5)
Supplement: Supplementary file 1 — Supplementary Information [file 41598_2019_44996_MOESM1_ESM.pdf]

Population divergence and gene flow in two East Asian shorebirds on the verge of speciation

Keren R. Sadanandan, Clemens Küpper, Gabriel W. Low, Cheng-Te Yao, Yue Li, Tao Xu, Frank E. Rheindt*, Shaoyuan Wu*

*corresponding authors

Supplementary information

Table S1: Summary of the 122 samples used, including specimen IDs, sampling localities and collection dates.

Sample ID	Country	Locality	Collection date (if available)
1283	China	Beihai	10 March 2004
1284	China	Beihai	10 March 2004
2895	China	Changle, Minjiang estuary	26 November 2004
2906	China	Changle, Minjiang estuary	27 November 2004
2908	China	Changle, Minjiang estuary	27 November 2004
2909	China	Changle, Minjiang estuary	27 November 2004
2910	China	Changle, Minjiang estuary	27 November 2004
2911	China	Changle, Minjiang estuary	27 November 2004
2912	China	Changle, Minjiang estuary	27 November 2004
NB10FZ	China	Fujian, Fuzhou	27 May 2015
NB12FZ	China	Fujian, Fuzhou	27 May 2015
NB13FZ	China	Fujian, Fuzhou	28 May 2015
NB14FZ	China	Fujian, Fuzhou	28 May 2015
NB15FZ	China	Fujian, Fuzhou	28 May 2015
NB18FZ	China	Fujian, Fuzhou	29 May 2015
NB20FZ	China	Fujian, Fuzhou	29 May 2015
NB21FZ	China	Fujian, Fuzhou	30 May 2015
NB22FZ	China	Fujian, Fuzhou	30 May 2015
NB23FZ	China	Fujian, Fuzhou	30 May 2015
NB24FZ	China	Fujian, Fuzhou	30 May 2015
NB38FZ	China	Fujian, Fuzhou	16 May 2016
NB39FZ	China	Fujian, Fuzhou	16 May 2016
NB40FZ	China	Fujian, Fuzhou	17 May 2016
NB41FZ	China	Fujian, Fuzhou	17 May 2016
NB42FZ	China	Fujian, Fuzhou	17 May 2016
NB43FZ	China	Fujian, Fuzhou	17 May 2016
NB45FZ	China	Fujian, Fuzhou	17 May 2016
NB46FZ	China	Fujian, Fuzhou	17 May 2016
NB47FZ	China	Fujian, Fuzhou	18 May 2016
NB48FZ	China	Fujian, Fuzhou	18 May 2016
NB49FZ	China	Fujian, Fuzhou	18 May 2016
NB9FZ	China	Fujian, Fuzhou	27 May 2015
NB11FZ	China	Fujian, Fuzhou	27 May 2015
NB16FZ	China	Fujian, Fuzhou	28 May 2015
NB17FZ	China	Fujian, Fuzhou	28 May 2015
NB19FZ	China	Fujian, Fuzhou	29 May 2015
NB44FZ	China	Fujian, Fuzhou	17 May 2016
NB8FZ	China	Fujian, Fuzhou	27 May 2015
12279	China	Fujian, Minjiang river	November 2009
12280	China	Fujian, Minjiang river	November 2009
12322	China	Fujian, Minjiang river	November 2009
NB25XM	China	Fujian, Xiamen	2 June 2015
NB27XM	China	Fujian, Xiamen	3 June 2015
NB28XM	China	Fujian, Xiamen	4 June 2015
NB29XM	China	Fujian, Xiamen	4 June 2015
NB30XM	China	Fujian, Xiamen	4 June 2015
NB31XM	China	Fujian, Xiamen	4 June 2015
NB50XM	China	Fujian, Xiamen	19 May 2016
NB51XM	China	Fujian, Xiamen	19 May 2016
NB52XM	China	Fujian, Xiamen	20 May 2016
NB53XM	China	Fujian, Xiamen	20 May 2016

NB54XM	China	Fujian, Xiamen	20 May 2016
NB26XM	China	Fujian, Xiamen	3 June 2015
779	China	Xinjiang, Luntai	11 September 2002
9736	China	Putian, Xinghua bay	20 January 2008
9737	China	Putian, Xinghua bay	20 January 2008
9738	China	Putian, Xinghua bay	20 January 2008
9739	China	Putian, Xinghua bay	20 January 2008
9740	China	Putian, Xinghua bay	20 January 2008
9741	China	Putian, Xinghua bay	20 January 2008
9743	China	Putian, Xinghua bay	20 January 2008
9744	China	Putian, Xinghua bay	20 January 2008
9745	China	Putian, Xinghua bay	20 January 2008
4421	China	Qinghai, Gangcha	13 August 2005
4432	China	Qinghai, Gangcha	13 August 2005
5551	China	Qinghai, Hada beach	20 April 2007
5554	China	Qinghai, Hada beach	21 April 2007
5555	China	Qinghai, Hada beach	21 April 2007
528	China	Qinhuangdao	24 August 2001
529	China	Qinhuangdao	24 August 2001
2678	China	Shanghai	-
3241	China	Yuanjiang, Dragon-Tiger forestry	19 December 2004
1435	China	Zhanjiang, Leizhou river	17 March 2004
1436	China	Zhanjiang, Leizhou river	17 March 2004
1437	China	Zhanjiang, Leizhou river	17 March 2004
NB1HZ	China	Zhejiang, Wenling	17 May 2015
NB2WL	China	Zhejiang, Wenling	19 May 2015
NB32WL	China	Zhejiang, Wenling	11 May 2016
NB33WL	China	Zhejiang, Wenling	12 May 2016
NB34WL	China	Zhejiang, Wenling	12 May 2016
NB4WL	China	Zhejiang, Wenling	21 May 2015
NB35WZ	China	Zhejiang, Wenzhou	13 May 2016
NB36WZ	China	Zhejiang, Wenzhou	13 May 2016
NB37WZ	China	Zhejiang, Wenzhou	14 May 2016
NB6WZ	China	Zhejiang, Wenzhou	23 May 2015
NB7WZ	China	Zhejiang, Wenzhou	24 May 2015
NB5WZ	China	Zhejiang, Wenzhou	23 May 2015
586	Japan	Chiba, Kisarazu	29 August 2004
238	Japan	Ibaraki, Kamisu	17 June 2007
239	Japan	Ibaraki, Kamisu	17 June 2007
446	Japan	Ibaraki, Kamisu	21 July 2008
450	Japan	Ibaraki, Kamisu	21 July 2008
842	Japan	Ibaraki, Kamisu	27 June 2009
843	Japan	Ibaraki, Kamisu	27 June 2009
888	Japan	Japan	30 June 2006
886	Japan	Okinawa	14 May 2007
590	Japan	Okinawa, Itoman	30 June 2006
601	Spain	Fuente de Piedra	-
602	Spain	Fuente de Piedra	-
603	Spain	Fuente de Piedra	-
604	Spain	Fuente de Piedra	-
605	Spain	Fuente de Piedra	-
606	Spain	Fuente de Piedra	-
607	Spain	Fuente de Piedra	-
1306	Taiwan	Changhua County	16 January 1998
1364	Taiwan	Changhua County	16 January 1998
1895	Taiwan	Changhua County	2003
2933	Taiwan	Changhua County	2004

7577	Taiwan	Changhua County	22 March 2008
2802	Taiwan	Hsinchu city	2003
2803	Taiwan	Hsinchu city	2003
2804	Taiwan	Hsinchu city	2003
2853	Taiwan	Hsinchu city	2003
2854	Taiwan	Hsinchu city	2003
2855	Taiwan	Hsinchu city	2003
2856	Taiwan	Hsinchu city	2003
2857	Taiwan	Hsinchu city	2003
6689	Taiwan	Nantou County	28 May 2008
1843	Taiwan	Taichung city	2003
5620	Taiwan	Taichung city	17 October 2007
6065	Taiwan	Taichung city	24 October 2007
3476	Taiwan	Taoyuan city	20 December 2003

Table S2: Summary of the field data collected during the 2015 and 2016 field seasons, including GPS coordinates and morphological measurements (all distance units in are in millimetres).

Sample ID	Location_GPS	Sex	Wing	Bill	Bill depth	Bill+head	Tarsus	Tail	Full length	Weight (g)
NB1	28°09'36.75" N; 121°19'05.54" E	F	116	15.80	3.50	42.64	28.30	49.0	-	41.54
NB2	28°09'36.75" N; 121°19'05.54" E	F	111	15.20	3.50	42.50	27.30	46.0	-	46.23
NB3	28°09'36.75" N; 121°19'05.54" E	F	117	15.30	3.60	41.90	26.30	50.0	-	44.54
NB4	28°09'36" N; 121°18'52"E	M	106	15.40	3.30	43.90	27.10	48.0	166	42
NB5	27°55'11"N; 120°56'1"E	F	110.5	18.20	3.30	44.00	28.80	54.0	175	50
NB6	27°55'11"N; 120°56'1"E	F	106	17.80	3.10	42.50	27.80	45.4	162	40
NB7	27°55'11"N; 120°56'1"E	M	107	17.10	3.50	43.20	26.10	41.6	162	45
NB8	26°1'24.1''N; 119°41'0.4''E	F	114	19.30	3.85	45.90	28.40	48.0	165	50
NB9	26°1'24.1''N; 119°41'0.4''E	F	116	18.85	4.32	45.80	28.30	48.0	165	53
NB10	26°1'24.1''N; 119°41'0.4''E	F	118.5	19.10	4.19	46.40	28.20	53.0	166	52
NB11	26°1'24.1''N; 119°41'0.4''E	M	115	20.02	4.14	46.80	28.60	47.0	163	48
NB12	26°1'24.1''N; 119°41'0.4''E	F	121	18.00	4.20	46.82	29.20	52.0	178	57
NB13	26°1'24.1''N; 119°41'0.4''E	F	116	18.50	4.20	47.10	29.11	50.0	172	54
NB14	26°1'24.1''N; 119°41'0.4''E	M	111	17.35	3.50	45.40	30.42	50.1	171	50

NB15	26°1'24.1''N; 119°41'0.4''E	F	115	17.85	3.78	44.18	28.10	54.0	166	51
NB16	26°1'24.1''N; 119°41'0.4''E	F	114	19.62	4.00	46.28	27.92	48.0	167	58
NB17	26°1'24.1''N; 119°41'0.4''E	F	111	19.65	3.60	45.43	29.50	46.0	165	62
NB18	26°1'24.1''N; 119°41'0.4''E	F	112	18.79	4.00	47.20	25.72	44.0	170	-
NB19	26°1'24.1''N; 119°41'0.4''E	F	118	19.45	3.51	46.35	29.00	53.8	175	50
NB20	26°1'24.1''N; 119°41'0.4''E	F	109	17.00	3.65	47.20	30.45	49.9	177	50
NB21	26°1'24.1''N; 119°41'0.4''E	F	109	17.00	3.80	42.50	26.20	51.2	162	47
NB22	26°1'24.1''N; 119°41'0.4''E	M	112	17.40	3.39	45.65	28.28	53.0	172	51.5
NB23	26°1'24.1''N; 119°41'0.4''E	F	108	17.70	3.75	45.70	28.24	43.0	170	64(with egg)
NB24	26°1'24.1''N; 119°41'0.4''E	M	111	19.81	3.10	45.30	28.00	47.0	157	45
NB25	24°33'28.0''N; 118°17'43.9''E	F	108	18.80	3.58	45.25	27.90	49.0	171	51
NB26	24°33'28.0''N; 118°17'43.9''E	chick	73	14.65	3.01	39.85	27.90	23.0	125	26
NB27	24°33'28.0''N; 118°17'43.9''E	F	111	17.50	3.70	44.72	28.90	45.0	168	47
NB28	24°33'28.0''N; 118°17'43.9''E	F	113	18.10	4.11	45.25	26.95	48.0	160	50
NB29	24°33'28.0''N; 118°17'43.9''E	M	117	19.22	4.20	46.81	28.65	49.0	160	53
NB30	24°33'28.0''N; 118°17'43.9''E	F	110	16.40	3.51	44.85	27.18	50.0	174	46
NB31	24°33'28.0''N; 118°17'43.9''E	M	110	17.90	3.52	44.32	29.63	51.0	175	53
NB32	28°9'16.02''N; 121°19'10.235''E	F	115	17.5	4.5	43.5	32	-	171	-
NB33	28°9'16.02''N; 121°19'6.383''E	F	112	16.5	5.1	43.1	29.5	45	169	-
NB34	28°9'16.02''N; 121°19'6.383''E	F	120	17.3	5	43.3	29.7	51	171	-
NB35	27°56'6''N; 120°58'1.523''E	F	112	17.1	4.7	43.9	27.3	42	166	-
NB36	27°56'6''N; 120°58'1.523''E	M	107	16.2	4.6	42.7	29.4	47	165	-
NB37	27°56'6''N; 120°58'1.523''E	M	106	16.5	4.9	43.3	30.5	41	168	-
NB38	26°1'24.1''N; 119°41'0.4''E	M	119	18.1	5.5	47.2	30.8	52	181	-
NB39	26°1'24.1''N; 119°41'0.4''E	M	115	17.9	4.8	46.6	28.9	47	175	-

NB40	26°1'24.1''N; 119°41'0.4''E	M	114	16.6	5.1	45.7	29.5	48	178	-
NB41	26°1'24.1''N; 119°41'0.4''E	M	115	17.6	4.9	46.4	29.9	52	183	-
NB42	26°1'24.1''N; 119°41'0.4''E	M	115	17.8	5.2	46.6	31.5	50	181	-
NB43	26°1'24.1''N; 119°41'0.4''E	F	115	17.2	5.2	43.1	28.7	48	174	-
NB44	26°1'24.1''N; 119°41'0.4''E	F	112	17.1	5	44.2	29.5	46	172	-
NB45	26°1'24.1''N; 119°41'0.4''E	F	119	18.7	5.5	46.7	29.3	49	180	-
NB46	26°1'24.1''N; 119°41'0.4''E	F	114	18	4.8	45.6	30.1	48	176	-
NB47	26°1'24.1''N; 119°41'0.4''E	F	111	18	5.3	46.1	29.1	48	179	-
NB48	26°1'24.1''N; 119°41'0.4''E	F	116	17.9	4.9	45	27.9	52	177	-
NB49	26°1'24.1''N; 119°41'0.4''E	M	116	18.6	5	47	28.9	50	182	-
NB50	24°32'39.516''N; 118°18'51.804''E	F	111	17.9	5.4	45.1	26.8	50	175	-
NB51	24°32'39.516''N; 118°18'51.804''E	F	112	17.1	5.6	46.9	28.9	48	172	-
NB52	24°32'39.516''N; 118°18'51.804''E	F	114	17.9	4.9	44.8	29.1	49	172	-
NB53	24°32'39.516''N; 118°18'51.804''E	M	115	-	5.2	44.5	23	50	175	-
NB54	24°32'39.516''N; 118°18'51.804''E	F	119	17.7	5.3	43.9	28.5	52	179	-

Table S3: Results of two-way ANOVA run on morphological measurements of genomically-prescribed Kentish and White-faced Plover groups. Significance codes are as follows: '***' 0.001 '**' 0.01 '*' 0.05

	**Wing**	**Bill**	**Bill_head**	**Bill_depth**	**Tarsus**	**Tail**	**Weight**	**Full_length**
Species	0.8676	0.0008***	0.0175*	0.6610	0.8725	0.1594	0.8400	0.2692
Sex	0.4993	0.4013	0.2019	0.9180	0.4757	0.7690	0.7810	0.6278
Species and sex	0.0646	0.5281	0.3433	0.4290	0.0029**	0.0366*	0.9140	0.0199*

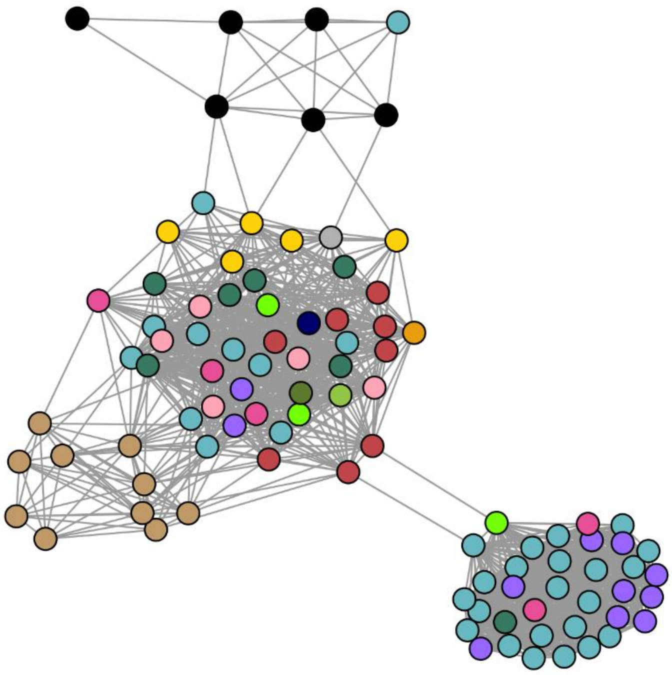

Figure S1: Kamada-Kawai force-directed depictions of mutual k-nearest neighbor network graphs for k = 35 show a network with two distinct clusters formed. Colours of circles reflect localities as per PCA diagram (Fig. 3), except Spain (depicted by black circles) and Xinjiang (depicted by gray circles).

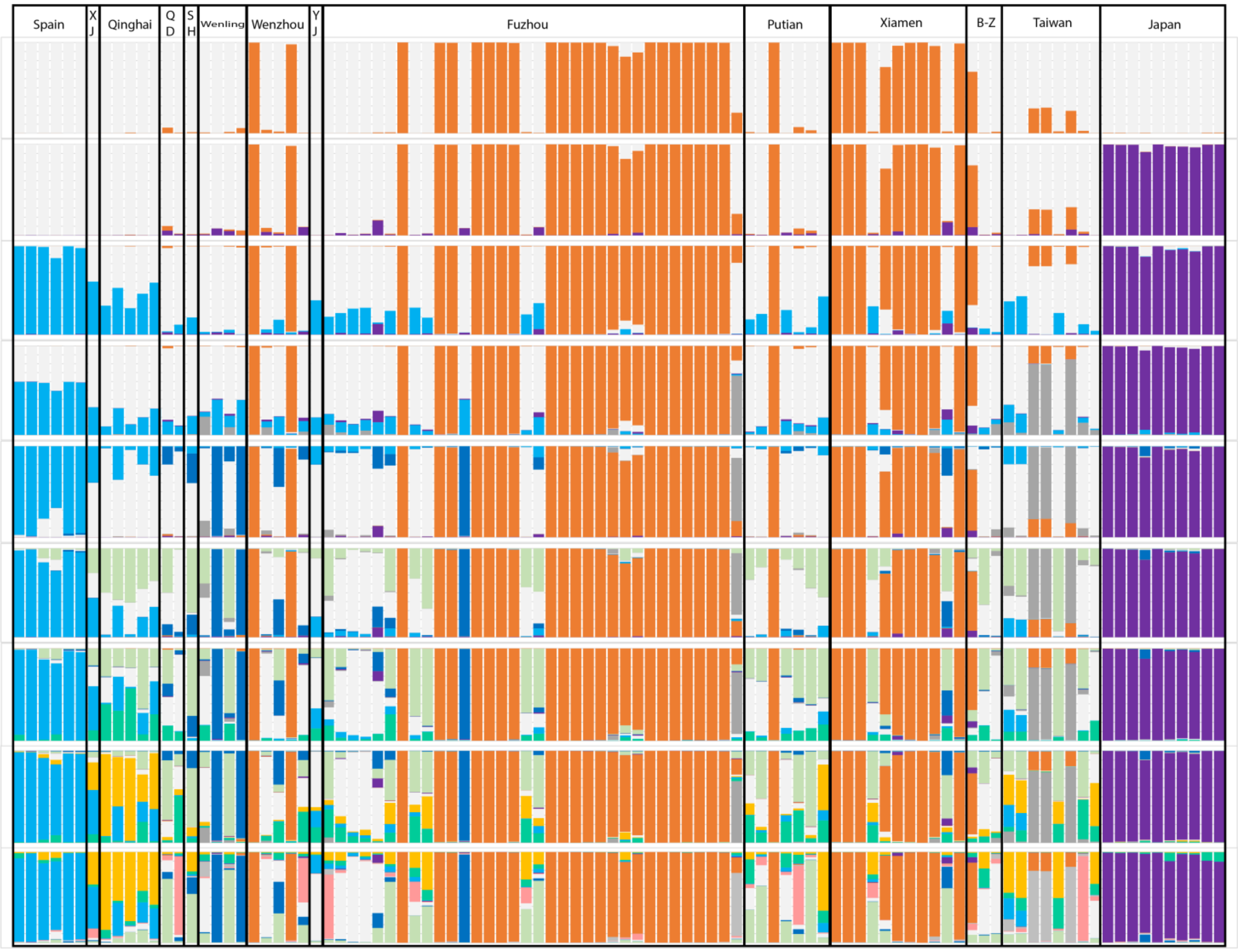

Figure S2: Averaged results of 5 iterations in STRUCTURE for *K* values from 2 (top) to 10 (bottom). Samples are grouped by sampling locality.
